# Evaluating the Role of *Anopheles* Mosquitoes in the Global Spread of Arboviruses: A Review of Laboratory-Confirmed Viral Competence

**DOI:** 10.3390/v18050541

**Published:** 2026-05-08

**Authors:** Rosheen Sungeni Mthawanji, Matthew Baylis, Maya Wardeh, Marcus S. C. Blagrove

**Affiliations:** 1 Institute of Infection, Veterinary and Ecological Sciences, University of Liverpool, Liverpool L69 7ZB, UK; 2 Department of Computer Science, University of Liverpool, Ashton Building, Liverpool L69 3DR, UK

**Keywords:** anopheles mosquitoes, arbovirus vector competence, virus overwintering

## Abstract

Mosquito-borne diseases are a major global health concern, infecting up to 700 million people annually and causing over one million deaths. Of the several genera of biting mosquitoes, species of *Anopheles* are mostly studied for their ability (vector competence) to transmit *Plasmodium* protozoan parasites, some species of which cause malaria. More than 480 species of *Anopheles* have been described worldwide, and about 70 of these are responsible for *Plasmodium* spp. transmission. However, the focus on *Anopheles* as vectors of *Plasmodium* has led to a relative lack of study about the ability of *Anopheles* to transmit viruses. Some *Anopheles* species have been experimentally confirmed as competent for various arboviruses. In most cases, they are secondary vectors, with relatively low competence, contributing to overall transmission while other species of mosquito or other vectors are responsible for sustained transmission. Although secondary vectors may contribute less to transmission, they may play important epidemiological roles by extending transmission seasons and/or providing a means of overwintering viruses. Here, we conducted a review of scientific repositories to build a database of known *Anopheles* competence for arboviruses. After exclusions, we retained 427 laboratory-confirmed studies from 7343 papers reviewed. Our analysis suggests some *Anopheles* spp. could contribute to arbovirus overwintering in temperate regions.

## 1. Introduction

Mosquito-borne viruses pose a global health threat due to their broad host range, infecting humans and animals annually. This poses a challenge for disease control experts as the inevitable spread to new locations and hosts complicate understanding vector dynamics [1]. During an arbovirus outbreak, the primary vector tends to be the focus of control programmes, whilst secondary vectors may remain under-characterized and insufficiently investigated [2]. An understanding of the importance of secondary vectors prior to outbreaks would increase the efficiency of control methods, redirect target response and mitigation procedures and anticipate transmission in regions or seasons where primary vectors are absent or inactive.

Among the hundreds of known arboviruses, only approximately 30 are known to cause human diseases. The Flaviviridae and Togaviridae viral families, particularly Japanese encephalitis (JEV), West Nile virus (WNV), dengue virus (DENV), and Zika virus (ZIKV), are commonly studied for causing severe clinical diseases. Alphaviruses in the Togaviridae family, such as Chikungunya (CHIKV), the encephalitic viruses (Eastern, Western, and Venezuelan), and the Ross River virus (RRV), are also of great public health importance [3]. Arbovirus transmission requires a competent vector: one capable of becoming infected and having the capability to then transmit the virus from one host to another. Important mosquito vectors include *Aedes aegypti*, *Aedes albopictus*, and *Culex quinquefasciatus*; these species are very widely distributed and are competent vectors of several arboviruses [2,4], but many other species are also known to be competent. However, many other species are also capable of transmitting viruses, and understanding the full range of vector competence is essential for effective disease control [5].

In tropical countries, arbovirus transmission tends to be year-round. For example, Dengue and Chikungunya virus transmission is sustained through mosquitoes being active all year, even though mosquito populations decline in the dryer months [6]. In this situation, competent secondary vectors could add to the overall burden, but the primary vectors are sufficient on their own to sustain transmission. However, different trends are seen in temperate regions, with viruses such as West Nile virus (WNV) and Usutu virus (USUV), as mosquitoes are only active for a smaller portion of the year [7,8]. There is an urgent need to understand the dynamics and methods around how the viruses persist in cold temperatures, when mosquitoes are not active. These may include vertical transmission [9] within mosquito populations, persistent infection in vertebrate hosts such as birds [6], or survival of infected mosquitoes through cold seasons. The evidence for long-term vertebrate infection for most arboviruses is limited, and vertical transmission has been confirmed for only a few arboviruses. The possibility that infected mosquitoes, particularly secondary vectors, could overwinter and reinitiate transmission in the spring remains underexplored.

Overwintering refers to the survival of organisms through adverse seasonal conditions, often via behavioural or physiological adaptations. In mosquitoes, this may involve diapause, a form of dormancy that allows survival through cold or dry periods. For example, many *Anopheles* mosquitoes’ diapause during temperature extremes and survive by using diapause to persist in long dry seasons in sub-Saharan Africa; instead of using their resources for reproduction, most processes halt and are allocated to longevity [10]. *Anopheles coluzzi* are known to diapause in harsh dry conditions while *Anopheles freeborni* diapause in extremely low temperatures [11]. Both species diapause in their adult forms.

The UK is a potential example of where viruses may persist in diapausing adults, as the same strain of USUV has persisted in the country for multiple years, despite the transmission season being only during the peak of summer [7]. Similar trends are seen in WNV overwintering in *Culex* sp. [12], and Blue Tongue Virus in Culicoides [13].

Here, we explore the possibility that *Anopheles* spp. could contribute to arbovirus overwintering by diapausing as infected (and ultimately infectious) adults. Even low levels of competence in a diapausing *Anopheles* spp. could significantly add to the risk of overwintering. Notably, African *Anopheles gambiae* and the Indo-Iranian *Anopheles stephensi* are well-studied due to the malaria-associated burdens in their respective regions [14]. To date, only one arbovirus of humans, the O’nyong-nyong virus, is known to be primarily transmitted by *Anopheles* mosquitoes in the field (*Anopheles funestus* and *An. gambiae*), although other *Anopheles* species have demonstrated the capability to be vectors of alphaviruses in laboratory settings and hence may be secondary vectors [15,16]. Understanding which mosquitoes are susceptible or capable of transmitting arboviruses is crucial for controlling threats to human and animal health.

To assess this possibility, we conducted a structured review of existing literature. Structured reviews provide a transparent and reproducible method for synthesizing evidence across diverse studies and are particularly valuable when addressing under-researched or fragmented topics.

Here we do this for:

(1) Laboratory *Anopheles* vector competence, and
(2) *Anopheles* diapausing habits to identify viruses in which overwintering in *Anopheles* potentially occur. Our aim is to identify arboviruses for which overwintering in Anopheles is plausible, and to assess how this could influence long-term persistence and seasonal transmission.

## 2. Materials and Methods

### 2.1. Data Collection

We conducted an extensive review of PubMed for relevant peer-reviewed studies. Our structured search employed multiple keywords:

“experimental transmission” OR “vector competence” OR “vector-competence” OR refractory OR “infection rate” OR “infection rates” OR “transmission rate” OR “transmission rates” OR “dissemination rate” OR “dissemination rates” AND mosquito.

The searches were limited to English-language publications. The term ‘mosquito’ was included in all queries. The review covered studies published between 1972 and 2023.

### 2.2. Exclusion/Inclusion Criteria

Duplicate records, systematic reviews, opinion pieces, modelling papers lacking experimental confirmation, and papers without vector competence data were excluded. Inclusion criteria restricted the dataset to laboratory studies of vector competence. Studies based solely on field inference were excluded to minimise confounding factors affecting infection outcomes.

Information extracted from the papers included the PubMed ID, title, digital object identifier (DOI), the virus of interest with its characteristics (virus class, name, tax ID, lineage, strain, accession number, and culture), mosquito details (species, tax ID, origin, location, lab colony or field-collected, generation, and collection coordinates), and experimental parameters (temperature, infectious dosage, unit used, feeding method, days post-infection, total infected, infection method, and transmission method). Experimental results, including the number infected, infection rate (proportion of mosquitoes with detectable virus after exposure), dissemination rate (proportion of infected mosquitoes in which the virus spread beyond the midgut), and transmission rate (proportion of mosquitoes with virus present in saliva or capable of transmitting it), were also recorded.

### 2.3. Screening and Data Verification

**Figure 1:**
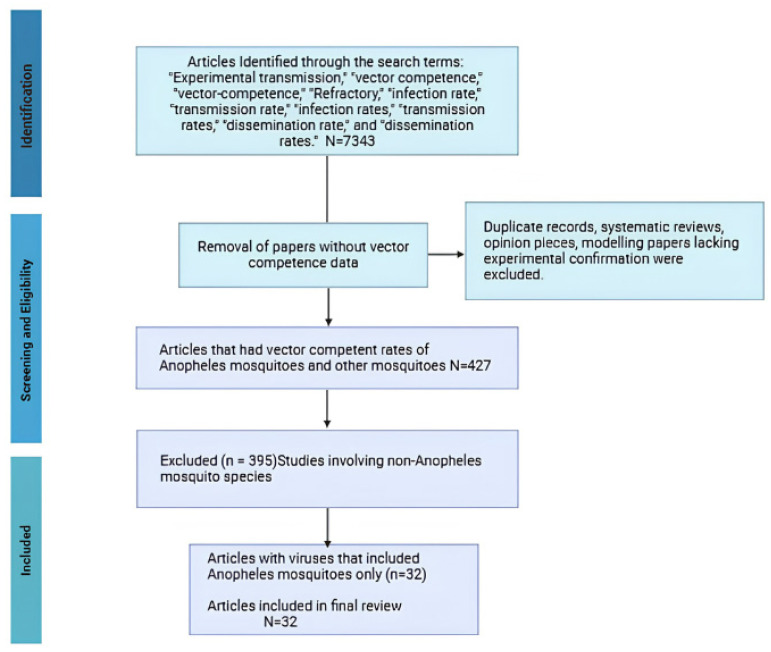
PRISMA Flowchart of main search strategy and article selection for paper selection.

We conducted a structured review to assess the role of *Anopheles* mosquitoes in arbovirus transmission. A total of 7343 articles were identified with our search terms. After removing records based on our above exclusion criteria, this screening yielded 427 articles reporting vector competence in *Anopheles* and other mosquito genera. All data were independently cross verified by two reviewers to ensure consistency and accuracy. The studies collectively covered 95 countries and involved 67 different viruses. For the purposes of this study, we extracted and analysed only the data related to *Anopheles* mosquitoes, all of which are included in the Supplementary Materials. These data are part of a larger, unpublished curated dataset compiling vector competence information across mosquito genera. The full process, including article selection and filtering criteria, is summarized in Figure 1.

## 3. Results

The vector competence data summarised in Supplementary Material Table S1 [13,14,17,18,19,20,21,22,23,24,25,26,27,28,29,30,31,32,33,34,35,36,37,38,39,40,41,42,43,44,45,46] shows findings from 32 studies examining *Anopheles* mosquitoes experimentally exposed to arboviruses in the laboratory. The studies were from 15 countries and presented five virus classes: alphavirus, orthoflavivirus, phlebovirus, orthobunyavirus, and alphamesonivirus and involved 15 *Anopheles* species, including *An. gambiae*, *An. stephensi*, *An. albimanus*, *An. quadrimaculatus*, and others. For each mosquito–virus pairing, we recorded data on infection, dissemination, and transmission. In cases where part of the experiment was not performed, it is registered as not done in the table (e.g., dissemination but not transmission was tested).

The results highlight variation in vector competence across virus families and mosquito species. Notably, several Anopheles species demonstrated high competence for alphaviruses such as Mayaro virus and O’nyong-nyong virus (ONNV), with consistently high infection, dissemination, and transmission rates. In comparison, most Anopheles species showed no competence for orthoflaviviruses such as dengue virus, Zika virus, and West Nile virus, which frequently yielded no infection or transmission. Some species, particularly *An. quadrimaculatus*, *An. stephensi*, and *An. gambiae*, also showed susceptibility to selected orthobunyaviruses (e.g., Cache Valley virus, CVV) and phleboviruses (e.g., Rift Valley fever virus, RVFV), although transmission rates varied.

It is important to note that most of the studies were conducted in the United States, reflecting a geographic bias in the available data and underscoring the urgent need for a deeper global investigation, particularly in endemic or emerging regions where *Anopheles* species are present. Altogether, this dataset shows the potential for *Anopheles* mosquitoes to act as secondary vectors for select arboviruses, especially in regions or seasons where primary vectors may be absent, inactive, not year-round, or less abundant.

**Figure 2:**
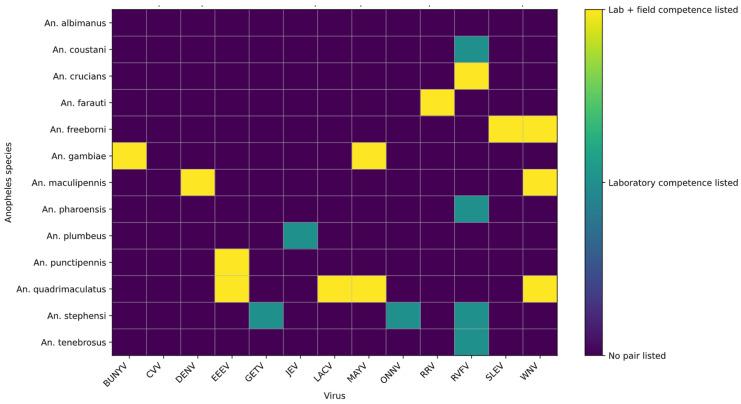
Anopheles species–arbovirus competence heatmap. Rows represent Anopheles mosquito species and columns represent arboviruses. Cells indicate laboratory-derived vector competence, reflecting progression from infection to dissemination and transmission, where available. This figure summarises experimentally tested mosquito–virus interactions and does not imply phylogenetic relationships, epidemiological significance, or transmission efficiency under natural conditions.

To examine experimentally tested mosquito–virus interactions, we visualised the relationships between *Anopheles* species and arboviruses included in our dataset. Figure 2 presents a summary of laboratory-based vector competence studies, highlighting which mosquito–virus pairings have been investigated and the extent of reported infection, dissemination, and transmission. Overall, the distribution of competence is uneven across both mosquito species and virus groups. Several *Anopheles* species, including *An. quadrimaculatus*, *An. gambiae*, and *An. freeborni*, show competence for multiple arboviruses, particularly alphaviruses such as Mayaro virus (MAYV), O’nyong-nyong virus (ONNV), and Sindbis virus (SINV), where infection, dissemination, and transmission have been reported. In contrast, competence for orthoflaviviruses, including dengue virus (DENV) and West Nile virus (WNV), appears more limited and species-specific, with relatively few positive interactions observed. Some species, such as *An. stephensi* and *An. pharoensis*, demonstrate laboratory evidence of competence for viruses including Rift Valley fever virus (RVFV), although progression to transmission is not consistently reported across studies. Notably, large areas of the heatmap remain unpopulated, reflecting a lack of experimental data rather than confirmed absence of competence.

However, the Orthoflaviviruses showed limited interactions with fewer mosquito species tested and mostly negative results, as seen in Supplementary Material Table S1 [13,14,17,18,19,20,21,22,23,24,25,26,27,28,29,30,31,32,33,34,35,36,37,38,39,40,41,42,43,44,45,46]. These viruses were primarily tested in *An. stephensi*, *An. gambiae*, and *An. albimanus.* Phleboviruses, including Rift Valley fever virus, were tested in several African and U.S. Anopheles species, while Orthobunyaviruses and Alphamesoniviruses had more restricted representation.

The patterns illustrated in Figure 2 emphasise both the potential and the limitations of current knowledge regarding *Anopheles*–arbovirus interactions. While several species demonstrate laboratory competence for multiple viruses, these interactions are highly uneven and remain poorly characterised across much of the genus. Notably, competence appears more frequently reported for alphaviruses, whereas evidence for orthoflaviviruses remains sparse and often negative, suggesting possible biological constraints or simply reflecting research bias.

Moderately high infection rates (>40%) were noted for 11 viruses: SINV, RVFV, JEV, ONNV, NGAV, MAYV, GETV, EIV, EEEV, CVV, and BUNYV. However, of the 11, eight were able to transmit. RVFV and GETV showed a transmission rate of over 50%.

Where competence was found, the possible role in the transmission of viruses was further investigated by reviewing diapause studies (Table 1)*. An. quadrimaculatus*, *An. freeborni*, *An. maculipennis*, *An. punctipennis*, *An. crucians* and *An. plumbeus*, have all shown vector competence in both field and laboratory settings, and these species also diapause as adults.

## 4. Discussion

While *Anopheles* mosquitoes are primarily known for malaria transmission [19,47], their potential role in arbovirus transmission and as overwintering vectors should not be overlooked. Comprehensive studies are required to understand the extent of this potential and its implications for public health. The results show that *Anopheles* mosquitoes are competent laboratory vectors to several viruses. We found that moderately high infection rates (>40%) were noted for 11 viruses: SINV, RVFV, JEV, ONNV, NGAV, MAYV, GETV, EIV, EEEV, CVV, and BUNYV (Supplementary Material Table S1 [13,14,17,18,19,20,21,22,23,24,25,26,27,28,29,30,31,32,33,34,35,36,37,38,39,40,41,42,43,44,45,46]). In the results, MAYV remains the most prevalent virus amongst most *Anopheles* species that have been tested; all tested *Anopheles* species were able to disseminate and transmit MAYV. RVFV was another common virus that showed high infection rates in *Anopheles*; however, most studies did not continue to test dissemination or transmission. These findings highlight that *Anopheles* mosquitoes, though not primary vectors, may contribute to secondary transmission cycles in regions where environmental or ecological conditions limit the activity of primary vectors.

The patterns illustrated in Figure 2 highlight both the potential and the limitations of current knowledge regarding *Anopheles*–arbovirus interactions. While several species demonstrate laboratory competence for multiple viruses, these interactions are unevenly distributed and remain poorly characterised across much of the genus. Competence appears more frequently reported for alphaviruses, whereas evidence for orthoflaviviruses is comparatively sparse and often negative, which may reflect biological constraints, experimental conditions, or underlying research bias. These gaps suggest that the current evidence base likely underestimates the true diversity of mosquito–virus interactions.

While other studies have summarized viruses found in *Anopheles* in both field and laboratory settings, this study specifically focuses on laboratory-confirmed vector competence and its potential implications [15]. Previous field studies support this possibility. At least 51 different viruses have been detected in *Anopheles* spp. in the field [47]. These studies have shown the potential to transmit diseases to both humans and other vertebrates. ONNV, SINV, RVFV, WNV, JEV and CVV are amongst some also seen in this study [16,19]. Collectively, this evidence suggests that *Anopheles* mosquitoes are not merely incidental hosts but may play ecologically meaningful roles in arbovirus maintenance.

A key finding from this review concerns the link between vector competence and diapause behaviour. Diapause allows mosquitoes to survive unfavourable environmental conditions by halting reproductive and metabolic processes [52]. Our synthesis shows that several *Anopheles* species capable of adult diapause including *An. quadrimaculatus*, *An. freeborni*, *An. maculipennis*, *An. punctipennis*, *An. crucians*, and *An. plumbeus* have also demonstrated laboratory or field competence for one or more arboviruses. This dual capability suggests a plausible mechanism for viral overwintering. While the potential for arbovirus persistence during diapause represents an important hypothesis, empirical evidence supporting viral survival and subsequent transmission following diapause remains limited. The extent to which arboviruses can persist without compromising mosquito survival, and whether replication resumes post-diapause, requires further investigation.

For instance, *An. freeborni* exhibits adult diapause in low temperatures in California [11], while *An. maculipennis* has been observed overwintering in bunkers in the Netherlands [53], co-occurring with *Culex pipiens* mosquitoes infected with Usutu virus (USUV). In that study, USUV persisted in overwintering *Culex* species, suggesting that similar mechanisms could occur in diapausing *Anopheles* populations. Given that *An. maculipennis* has demonstrated high competence for WNV and DENV in laboratory studies [54], this raises the possibility that arboviruses could persist through winter within *Anopheles* populations in temperate zones.

This potential overwintering mechanism would allow transmission cycles to persist year-to-year, even when primary vector populations decline during cold seasons. Such persistence could have major epidemiological implications, particularly in temperate regions where viral activity typically resumes each summer.

Further experimental and modelling work is required to clarify the contribution of *Anopheles* species to arbovirus overwintering. For example, *An. stephensi*, which does not diapause, remains active year-round in certain regions and has demonstrated competence for JEV, GETV, RVFV, and ONNV [11,24]. Such species could act as continuous transmission reservoirs, whereas diapausing species might support virus persistence during inactive seasons.

Future modelling should incorporate the overwintering potential of *Anopheles* vectors into risk prediction frameworks. Doing so could extend the estimated range of arbovirus risk further polewards and increase the projected scale of annual outbreaks, as viruses may already be present in the environment when the transmission season begins, rather than requiring reintroduction each year.

An additional but important consideration is the potential interaction between *Plasmodium* infections and arboviruses within *Anopheles* mosquitoes. Immune responses activated during *Plasmodium* infection, including pathways such as nitric oxide production, may influence viral susceptibility, while viral infections may in turn affect parasite development [55]. These within-vector interactions remain poorly understood but are likely to play a critical role in shaping transmission dynamics in regions where malaria and arboviruses co-circulate.

### Limitations of Current Evidence

A key limitation of the current evidence base is the heavy reliance on laboratory studies to assess vector competence. Unlike in the field where temperature and humidity are variable, experimental infections conducted in the laboratory are performed under strict conditions that are controlled, and the viral titers used are extremely higher than those encountered under natural settings, which may not reflect transmission dynamics. Furthermore, vector competence does not always translate into vectorial capacity, there are other factors like host preference, mosquito lifespan, etc., that play a role in epidemiology.

Geographical distribution of available studies highlights the USA as the place where most of the studies are conducted. This may limit the generalizability of findings to where Anopheles diversity is highest.

Finally, Virus interactions with other pathogens remain understudied in Anopheles, so interpretation of laboratory competence should be taken with caution.

## 5. Conclusions

Our review suggests that *Anopheles* mosquitoes, traditionally viewed as vectors of Plasmodium parasites, are also experimentally competent for several arboviruses, including O’nyong-nyong virus (ONNV), Mayaro virus (MAYV), Rift Valley fever virus (RVFV), and others. Although competence levels are generally lower than in *Aedes* or *Culex* species, the evidence indicates that *Anopheles* mosquitoes can act as secondary or supporting vectors. Their potential contribution to virus maintenance, particularly through overwintering mechanisms, has likely been underestimated.

The association between vector competence and diapause suggests that *Anopheles* mosquitoes may play a previously unrecognised role in viral persistence during cold or dry seasons. Diapause enables adult mosquitoes to survive in temperate regions, potentially allowing virus survival between transmission seasons. Even if infection rates are low, the prolonged lifespan of diapausing mosquitoes could extend the window for virus maintenance and early-season re-emergence. Ultimately, recognising *Anopheles* mosquitoes as potential secondary arbovirus vectors adds a crucial dimension to vector surveillance and global disease control strategies.

## Figures and Tables

**Table 1:** Mosquito diapause capability, vector competence demonstrated in laboratory or field settings, and the viruses for which they are competent, and references.

Mosquito Species	Diapause as Adults?	Competence Shown in the Field or Lab	Virus	Reference
*Anopheles farauti*	no	field	RRV	[40]
*Anopheles stephensi*	no	laboratory	RVFV, GETV, ONNV	[11,18]
*Anopheles quadrimaculatus*	Yes	both	LACV, EEEV, WNV, MAYV	[42]
*Anopheles freeborni*	Yes	both	WNV, SLEV	[15,47]
*Anopheles gambiae*	no	both	MAYV, BUNYV	[15,47]
*Anopheles maculipennis*	Yes	both	DENV, WNV	[15,48,49,50]
*Anopheles pharoensis*	Yes	laboratory	RVFV	[18,47,51]
*Anopheles tenebrosus*	Yes	laboratory	RVFV	[18,47,51]
*Anopheles punctipennis*	Yes	both	EEEV	[31,32,47,51]
*Anopheles coustani*	no	laboratory	RVFV	[29]
*Anopheles bradleyi*	Yes	laboratory	RVFV	[18,51]
*Anopheles crucians*	Yes	both	RVFV	[18,32,33,51]
*Anopheles plumbeus*	Yes	laboratory	JEV	[40,44,52]

## Data Availability

The data presented in this study are available from the corresponding author upon reasonable request.

## Supplementary Materials

The following supporting information can be downloaded at: https://www.mdpi.com/article/10.3390/v18050541/s1, Table S1: Data extracted from the Anopheles’ data showing virus classification, mosquito species, Country of origin, vector competence and references [13,14,17,18,19,20,21,22,23,24,25,26,27,28,29,30,31,32,33,34,35,36,37,38,39,40,41,42,43,44,45,46].

## Abbreviations

The following abbreviations are used in this manuscript:

BUNYV	Bunyamwera virus
CHIKV	Chikungunya
CVV	Cache Valley Virus
DENV	Dengue virus
EEEV	Eastern Equine Encephalitis virus
GETV	Getah Virus
JACV	James Canyon virus
JEV	Japanese Encephalitis virus
LACV	La Crosse virus
MAYV	Mayaro virus
ONNV	O’nyong-nyong virus
RRV	Ross River virus
RVFV	Rift Valley fever virus
SINV	Sindbis virus
SLEV	St. Louis encephalitis virus
USUV	Usutu virus
WNV	West Nile Virus
ZIKV	Zika virus
