# Supplementary material for: Evaluating the Role of Anopheles Mosquitoes in the Global Spread of Arboviruses: A Review of Laboratory-Confirmed Viral Competence
Source: Viruses. 2026 May 8;18(5):541. doi: 10.3390/v18050541 (PMC13211685; doi:10.3390/v18050541)
Supplement: Supplementary file 1 [file viruses-18-00541-s001.zip › viruses-4300655-supplementary.pdf]

Supplementary Material

Table S1. Data extracted from the *Anopheles'* data showing virus classification, mosquito species, Country of origin, vector competence and references. [supplementary material], low percentage values were defined as those ranging from 0% to 40%, whereas high percentage values were defined as those from 50% and above.

**Virus Class**	**virus**	**mosquito species**	**mosquito origin [country]**	**Infection**	**Dissemination**	**Transmission**	**Reference**
Alphavirus	Onyong-nyong virus	*Anopheles stephensi*	Pakistan	yes, high	yes, high	yes, high	[14]
Alphavirus	Getah virus	*Anopheles stephensi*	Japan	Yes	yes	yes	[17]
Orthoflavivirus	West Nile virus	*Anopheles farauti*	Australia	No	no	no	[18]
Alphavirus	Mayaro virus	*Anopheles albimanus*	United States	yes, high	yes	yes	[19,20,21]
Alphavirus	Sindbis virus	*Anopheles albimanus*	United States	yes, high	yes	yes	[17,20]
Alphavirus	Chikungunya virus	*Anopheles albimanus*	United States	yes, low	yes, low	yes, low	[20]
Orthoflavivirus	Dengue virus 2	*Anopheles albimanus*	United States	No	not done	not done	[20]
Orthobunyavirus	Cache Valley virus	*Anopheles quadrimaculatus*	United States	yes high	yes	yes	[22]
Alphavirus	Mayaro virus	*Anopheles freeborni*	United States	yes, low	yes	yes	[21]
Alphavirus	Mayaro virus	*Anopheles gambiae*	United States	yes, high	yes	yes	[21]
Alphavirus	Mayaro virus	*Anopheles quadrimaculatus*	United States	yes, high	yes	yes	[21]
Alphavirus	Mayaro virus	*Anopheles stephensi*	United States	yes, high	yes	yes	[19,20,21]
Orthoflavivirus	Dengue virus 2	*Anopheles maculipennis*	Italy	not done	no	no	[23]
Alphamesonivirus	Dianke virus	*Anopheles gambiae*	Senegal	yes, high	yes, low	no	[21]
Orthobunyavirus	Jamestown Canyon virus	*Anopheles quadrimaculatus*	United States	yes, high	yes, low	no	[24]
Orthoflavivirus	Zika virus	*Anopheles gambiae*	United States	no	no	no	[25]
Phlebovirus	Rift Valley fever virus	*Anopheles pharoensis*	Egypt	yes, high	yes, low	not done	[26]
Phlebovirus	Rift Valley fever virus	*Anopheles tenebrosus*	Egypt	yes, high	yes, low	not done	[26]
Alphavirus	Mayaro virus	*Anopheles quadrimaculatus*	United States	yes, high	yes, high	yes, low	[27]
Alphavirus	Eastern equine encephalitis virus	*Anopheles punctipennis*	United States	yes, high	not done	no	[28]
Alphavirus	Eastern equine encephalitis virus	*Anopheles quadrimaculatus*	United States	yes, high	not done	no	[28]
Phlebovirus	Rift Valley Fever virus	*Anopheles stephensi*	Netherlands	yes, low	yes, low	yes, low	[13]
Phlebovirus	Rift Valley fever virus	*Anopheles coustani*	Madagascar	yes, low	yes, low	yes, low	[29]

Phlebovirus	Rift Valley fever virus	*Anopheles gambiae*	Cameroon	not done	yes, low	not done	[30]
Phlebovirus	Rift Valley fever virus	*Anopheles bradleyi-crucians*	United States	yes, high	yes, low	not done	[31]
Phlebovirus	Rift Valley fever virus	*Anopheles crucians*	United States	yes, high	not done	not done	[32]
Phlebovirus	Rift Valley fever virus	*Anopheles quadrimaculatus*	United States	yes, high	no	no	[33]
Orthoflavivirus	Zika virus	*Anopheles quadrimaculatus*	United States	no	no	no	[34]
Sunrhavirus	Sunguru Virus	*Anopheles gambiae*	Uganda	yes, low	not done	not done	[35]
Orthobunyavirus	Bunyamwera virus	*Anopheles gambiae*	Kenya	yes, high	yes, high	not done	[36]
Orthobunyavirus	Cache Valley virus	*Anopheles quadrimaculatus*	United States	yes, high	yes, high	yes, low	[37]
Orthoflavivirus	Usutu virus	*Anopheles plumbeus*	France	yes, low	yes, low	no	[38]
Alphavirus	Mayaro virus	*Anopheles gambiae*	NA	yes, high	yes, high	no	[39]
Orthoflavivirus	Japanese Encephalitis virus	*Anopheles plumbeus*	Belgium	yes, high	yes, low	yes, low	[40]
Alphavirus	Onyong-nyong virus	*Anopheles gambiae*	NA	yes, high	not done	not done	[41]
Alphavirus	Eastern equine encephalitis virus	*Anopheles quadrimaculatus*	United States	yes, high	yes, high	no	[42]
Alphavirus	Eilat virus	*Anopheles gambiae*	NA	yes, high	not done	no	[43]
Orthoflavivirus	Dengue virus 4	*Anopheles stephensi*	Taiwan	no	not done	not done	[44]
Alphavirus	Onyong-nyong virus	*Anopheles gambiae*	NA	yes, high	not done	not done	[45]
Alphavirus	Eilat virus	*Anopheles gambiae*	NA	yes, high	yes, low	not done	[46]
